# Role and application of stem cells in dental regeneration: A comprehensive overview

**DOI:** 10.17179/excli2021-3335

**Published:** 2021-02-22

**Authors:** Armin Soudi, Mohsen Yazdanian, Reza Ranjbar, Hamid Tebyanian, Alireza Yazdanian, Elahe Tahmasebi, Ali Keshvad, Alexander Seifalian

**Affiliations:** 1 Research Center for Prevention of Oral and Dental Diseases, Baqiyatallah University of Medical Sciences, Tehran, Iran; 2 Department of Veterinary, Science and Research Branch, Islamic Azad University, Tehran, Iran; 3 Nanotechnology and Regenerative Medicine Commercialization Centre (Ltd), The London Bioscience Innovation Centre, London, UK

**Keywords:** dental, stem cells, regeneration, stem cell therapy

## Abstract

Recently, a growing attention has been observed toward potential advantages of stem cell (SC)-based therapies in regenerative treatments. Mesenchymal stem/stromal cells (MSCs) are now considered excellent candidates for tissue replacement therapies and tissue engineering. Autologous MSCs importantly contribute to the state-of-the-art clinical strategies for SC-based alveolar bone regeneration. The donor cells and immune cells play a prominent role in determining the clinical success of MSCs therapy. In line with the promising future that stem cell therapy has shown for tissue engineering applications, dental stem cells have also attracted the attention of the relevant researchers in recent years. The current literature review aims to survey the variety and extension of SC-application in tissue-regenerative dentistry. In this regard, the relevant English written literature was searched using keywords: “tissue engineering”, “stem cells”, “dental stem cells”, and “dentistry strategies”. According to the available database, SCs application has become increasingly widespread because of its accessibility, plasticity, and high proliferative ability. Among the growing recognized niches and tissues containing higher SCs, dental tissues are evidenced to be rich sources of MSCs. According to the literature, dental SCs are mostly present in the dental pulp, periodontal ligament, and dental follicle tissues. In this regard, the present review has described the recent findings on the potential of dental stem cells to be used in tissue regeneration.

## Abbreviations

2D: Two-Dimensional 

3D: Three-Dimensional 

Ab: Antibody 

AM: Adrenomedullin 

APA: Alkaline Phosphatase Activity 

ASC: Adult Stem Cell 

BBB: Basso Beattie and Bresnahan scores 

BMMSCs: Bone Marrow-derived Mesenchymal Stem Cells 

BMPs: Bone Morphogenetic Proteins

BMP7: Bone Morphogenetic Protein-7

BMS-345541: 4(2'-aminoethyl) amino-18-dimethylimidazo(12-a)quinoxaline 

BMSCs: Bone Marrow-derived MSCs

Ca(OH)_2_: Calcium Hydroxide-Based Materials 

CDMSCs: Craniofacial-Derived Mesenchymal Stem Cells 

CIA: Collagen-Induced Arthritis 

CK: Cytokeratin 3 

CRE: cAMP Response Elements 

CREB: cAMP Response Element-Binding

DBCs: Dental Bud Cells 

DC: Dendritic Cell 

DESCs: Dental Epithelial Stem Cells

DEXA: Dual-Energy X-Ray Absorptiometry 

DFSCs: Dental Follicle Stem Cells 

DPPSC: Dental Pulp Pluripotent-like Stem Cells 

DPSC: Dental Pulp Stem Cells 

hDPSCs: Human Dental Pulp Stem Cells

DSC: Dental Stem Cell 

DSPP: Dentin Sialophosphoprotein 

EAE: Experimental Autoimmune Encephalomyelitis 

ECM: Extracellular Matrix

EGF: Epidermal Growth Factor 

EMD: Enamel Matrix Derivative 

ESCs: Embryonic Stem Cells 

FDA: US Food and Drug Administration

GDF: Growth/Differentiation Factor 

GDNF: Glial Cell Line-Derived Neurotrophic Factor 

GMSCs: Gingiva-derived Mesenchymal Stem Cells 

HA: Hydroxyapatite 

HBGF: Heparin-Binding Growth Factor 

hDPC: Human Dental Pulp Cells 

hDPSC: Human Dental Pulp Stem Cells

HGF: Hepatocyte Growth Factor 

HI: Hypoxia-Ischemia 

HSCs: Hematopoietic Stem Cells 

HUVECs: Human Umbilical Vein Endothelial Cells 

ICCs: Islet-Like Cell Clusters 

IDCs: Immature Dendritic Cells 

IDO: Indoleamine 23-Dioxygenase 

IFN: Interferon 

IGF: Insulin-like Growth Factors 

IKK: IκB Kinase 

IPAPCs: Inflamed Periapical Progenitor Cells 

iPSCs: Induced Pluripotent Stem Cells

ISCT: International Society for Cellular Therapy 

JBMMSCs: Jaw Bone Marrow Mesenchymal Stem Cells 

LLLI: Low-Level Laser Irradiation 

LOXL2: Lysyl Oxidase-Like 2 

MAPK: Mitogen-Activated Protein Kinase 

MSCs: Mesenchymal Stem Cells 

MTA: Mineral Trioxide Aggregate

NCPs: Non-collagenous Proteins 

NETs: Neutrophil Extracellular Traps 

NGS: Next-Generation Sequencing 

PBMC: Peripheral Blood Mononuclear Cell 

PDGF: Platelet-Derived Growth Factor 

PDLSCs: Periodontal Ligament Stem Cells 

PGE2: Prostaglandin E2 

pLN: Pancreatic Lymph Nodes 

PRF: Platelet-Rich Fibrin 

PRP: Platelet Rich Plasma 

PTH: Parathyroid Hormone 

QCT: Quantitative Computed Tomography 

rh-PDGF: Recombinant Human Platelet-Derived Growth Factor 

ROCK: Rho-Associated Coiled-Coil Containing Protein Kinase 

ROS: Reactive Oxygen Species 

RTKs: Receptor Tyrosine Kinase Cascades 

RT-PCR: Reverse Transcription-Polymerase Chain Reaction 

SAOS-2: Sarcoma Osteogenic Cell Line 

SCAP: Stem Cell from Apical Papilla 

SCI: Spinal Cord Injury 

SDF1: Stromal-Derived Factor-1 

SGSCs: Salivary Gland Stem Cells 

SHED: Stem Cells from Human Exfoliated Deciduous Teeth 

STZ: Streptozotocin 

T1DM: Type 1 Diabetes 

TBI: Traumatic Brain Injury 

TCP: Tricalcium Phosphate 

TGFs: Transforming Growth Factors 

TGF‐β: Tumor Growth Factor β 

TLR-4: Toll-Like Receptor-4 

TMJ: Temporomandibular Joint 

TNF-α: Tumor Necrosis Factor-α 

VEGF: Vascular Endothelial Growth Factor. 

## Introduction

Any trauma, disease, or congenital abnormalities that lead to tissue loss in the craniofacial region that affect the aesthetic and/or function of the craniofacial area, can raise severe physiological and psychological sequela for the patients (Zaky and Cancedda, 2009[145]). Losing alveolar bone for any reason is also a challenge for clinicians since conventional treatments such as dentures or implants usually do not achieve enough satisfactory outcomes that content patients (Mitsiadis et al., 2017[82]), mainly because the implanted or denture teeth are weaker than the natural tooth (Feng et al., 2016[30]). Significant bone tissue loss in the craniofacial area frequently occurs due to periodontal disease, congenital abnormalities, tumors, traumatic injury, or resorption secondary to tooth loss (Mani et al., 2014[76]). In general, the reconstruction strategies are extended from using medical devices to tissue grafts and/or tissue engineering approaches. This review mainly addresses the researches regarding the modern methods for orofacial reconstruction approaches that use stem cells for tissue engineering. These innovative methods utilize specific bioactive/biodegradable synthetic or natural scaffolds often together with advanced molecular techniques to return the function and appearance to the damaged tissue as much as possible. Here, the scaffold types and methodologies which are shown in the literature that enable cells to produce the efficient extracellular matrix (ECM) are briefly implied. An efficient ECM is desired to ultimately convert to a functional tissue with eligible geometry, size, and composition. By looking at the proceeding of regenerative medicine, it can be notably observed that the medical devices and whole-tissue grafts are increasingly replaced by the tissue regenerative engineering approaches. These neoteric strategies include using specific materials as scaffolds (sometimes combined with certain molecules) for growing compatible cells to substitute the diseased or damaged tissue with a functional one in-situ (Zaky and Cancedda, 2009[145]). Dentistry has passed several eras each of which is distinguished by trending different materials and methodologies. The most recent progress in dentistry is distinguished by the extensive attempts to use biomaterials for replacing injured craniofacial tissue with a functional natural-like tissue (Abou Neel et al., 2014[2]). The first predominantly used materials were metal implants and associated devices (the 1950s) whose effects on the surrounding tissues and cells resulted in their substitution with more biocompatible polymers and synthetic materials ('70s and '80s) (Yan et al., 2010[141]). Concurrently with the extending surgical-free material-based reconstruction approaches in clinical prosthodontics, stem cell (SC)-based regeneration and associated therapies in periodontal diseases started developing too (Wang et al., 2010[135]; Yan et al., 2010[141]). The modern SC-based regenerative medicine has facilitated various reconstruction therapies in clinical implant dentistry such as regeneration (Yan et al., 2010[141]). However, most studies manipulate SCs in *ex-vivo* conditions using different physical matrices (Wang et al., 2010[135]). One of the most state-of-the-art dental material studies has focused on designing and using natural and degradable biologic-based materials as scaffolds for regenerating periodontal tissues *in-vivo* (Wang et al., 2010[135]; Abou Neel et al., 2014[2]). For this purpose, the required stem cells have been obtained from different sources, including bone marrow (BM), periodontal ligament (PDL), etc., and have been applied with different types of bone grafts such as autografts, xenografts, allografts, and alloplastic materials (Wang et al., 2010[135]). It cannot be concluded yet from the current literature that which donor sources provide the most appropriate cell isolation (Wang et al., 2010[135]). SC-based approaches have developed to the point that makes it possible to replace the missing teeth with bioengineered ones that have already brought the dental stem cell (DSC)-banking for future regenerative uses to the market (Egusa et al., 2012[24]). In this regard, understanding the fundamentals of SCs and their associated technologies seems to be necessary for dentistry clinicians and relevant-fields' researchers (Yan et al., 2010[141]). Accordingly, the current study has critically reviewed the applications of stem cells in reconstructive dentistry.

## Stem Cell Types and Sources

The SC types ever investigated for application in regenerative medicine can be divided into two categories: embryonic stem cells (ESCs) and adult stem cells (ASCs). ESCs are pluripotent stem cells originating from the inner cell mass of the blastocyst-stage embryos (Mahla, 2016[74]; Hu et al., 2018[45]). They can differentiate into almost all specific lines. Whereas, ASCs are typically categorized as non-pluripotent cells, instead, as multipotent stem cells that exist in few numbers within adult tissues and are responsible for maintaining tissues healthy and repairing damages by self-regeneration and differentiation into specific cell types (Paz et al., 2018[107]). ASCs are also known as somatic stem cells or postnatal stem cells and can be isolated from various adult organs, including bone, muscle, skin, nerve, pancreas, heart, and dental tissues (Mahla, 2016[74]). Furthermore, multiple adult SC lines can now be induced to be reprogrammed and produce induced pluripotent stem cells (iPSCs) (Paz et al., 2018[107]) recalled as plasticity potential (Towns and Jones, 2004[128]). The first stem cells used in regenerative medicine applications were isolated from bone marrow; however, today, it is demonstrated that the unspecialized cells called “stem cells” present not only in the bone marrow but also in many other tissues and organs, including dental pulp cells (Potdar and Deshpande, 2013[111]). The postnatal dental stem cells are primarily originated from either epithelial cells or mesenchymal cells (Lymperi et al., 2013[70]). Likely, the only niche for the epithelial dental SCs is recognized to be in the apical end of rodents' incisors (Paz et al., 2018[107]). The mesenchymal dental SCs can be derived from different sources, including bone marrow and non-marrow tissues from either extra-oral or intra-oral niches. The bone marrow-derived stem cells (BMSCs) used for regenerating dental tissues are generally isolated from extra-oral origins (femur and iliac crest) or orofacial bones (maxilla and mandible bone marrow) obtained through dental treatments. Despite the early positive outcome of autologous craniofacial bone grafting, there are some drawbacks and challenges such as the invasive isolation method of extra-oral BMSCs and lower alternative sources of dental stem cells (Abdel Meguid et al., 2018[1]; Hu et al., 2018[45]; Paz et al., 2018[107]). Therefore, several mesenchymal stem cells are introduced in the literature from non-marrow orofacial or extra-oral sources such as; stem cells of the primary tooth (SHEDs), stem cells of apical papilles (SCAPs), stem cells of periodontal ligament (PDLSCs), and precursor cells of the dental follicle (DFPCs). In the regenerative medicine, the key products in successful outcomes are not only stem cells, but also the 3D scaffold, growth factors for differentiation and proliferation, as well as bioreactors. The highlights of recent researches on the oral stem cells are summarized in Table 1(Tab. 1) (References in Table 1: Chrepa et al., 2017[17]; De Berdt et al., 2015[22]; Farhad Mollashahi et al., 2016[27]; Ferro et al., 2011[31]; Gao et al., 2013[32]; Ginani et al., 2018[34]; Gu and Shi, 2016[37]; Gu et al., 2013[36]; Huang et al., 2017[46]; Iwasaki et al., 2014[49]; Jeong et al., 2013[52]; Kanafi et al., 2013[55]; Kim et al., 2013[58]; Kim et al., 2015[57]; Kim et al., 2017[56]; Lertchirakarn and Aguilar, 2017[63]; Li et al., 2017[65]; Liu et al., 2018[68]; Maher et al., 2018[73]; Maimets et al., 2015[75]; Martinez-Sarra et al., 2017[78]; Nicola et al., 2017[92]; Nunez-Toldra et al., 2017[96], 2019[97]; Peng et al., 2017[108]; Pringle et al., 2016[113]; Regmi et al., 2017[114]; Rossato et al., 2017[117]; Sanches et al., 2018[119]; Tsai et al., 2015[129]; Ustiashvili et al., 2014[133]; Xu et al., 2017[139]; Yuan et al., 2015[143]; Zhang et al., 2009[149], 2017[152], 2019[151]).

## Embryonic Stem Cells

The embryonic stem cell (ESC) is a more general terminology for pluripotent human embryonic stem (hES) cells with stem cell-like developmental quality *in-vitro* (Zaky and Cancedda, 2009[145]). The hES cells show three properties which profound them as a qualified platform for developing an extensive range of cell types; 1) hES cells surprisingly remain in the second week of development within *in-vivo* niches, 2) they are much higher scalable in the undifferentiated state compared to other SC types (Zaky and Cancedda, 2009[145]), 3) they can be clonally isolated probably due to the presence of specific transcription factors (homeobox genes); however, the exact mechanism and the uniformity of these genes have remained to be studied in more details (Gebhard et al., 2007[33]). Findings from animal model studies and cellular/molecular studies suggest that the clonal derivation of hES cells *in-vitro* might be similar to what happens for neural crest cells when their fate is specified before migration to construct the mesenchyme of embryonic branchial arches structures (Zaky and Cancedda, 2009[145]; Sternberg et al., 2012[122]). The clonal isolation potential of hES cells is essential for producing a wide range of stem cells in vitro for research and therapy purposes (Sternberg et al., 2012[122]). While other SC types differentiate into heterogeneous cell lines that should be purified and may become problematic by producing unwanted cell lines that grow to ectopic tissues in the graft (Sternberg et al., 2012[122]). Previously, the common method for restricting undefined cell types has been using clonal embryonic progenitor cell lines from available cell banks. This method contaminates the designed grafts for clinical applications (Sternberg et al., 2012[122]). Using clonal embryonic SC lines leads to more scalability and less lot-to-lot variability that allows repeating fundamental experiments on the same lines and facilitate producing more site-specific tissues to be applied in regenerative clinical therapies (Sternberg et al., 2012[122]).

## Dental Pulp Pluripotent-Like Stem Cells

Dental pulp pluripotent stem cells (DPPSCs or DPSCs) originate from the cranial neural crest in the embryonic stage (Zhang et al., 2017[148]). The isolation of dental stem cells can be done by size-sieved isolation, stem cell colony cultivation, magnetically activated cell sorting (MACS), and fluorescence-activated cell sorting (FACS). In addition, the preservation of dental stem cells can be categorized as cryopreservation and magnetic freezing (Bansal and Jain, 2015[5]). Markers that can detect DPSCs are STRO-1 and CD146 (Yang et al., 2018[142]; Zhai et al., 2018[146]). DPSCs are invasively obtained from the third molar (wisdom teeth) with less ethical concerns and show favorable MSC-like characteristics such as multipotency and self-renewal procedures (Caseiro et al., 2016[11]). DPSCs are unique in lineage differentiation as they express neuron-related markers before developing into functional neuron-like cells with the ability to produce neurotrophic factors such as neurotrophin (NT), which makes them promising candidates for SC-based nerve regeneration therapies (Ullah et al., 2017[131]). Differentiating DPSCs into neurons have been experimentally induced through various protocols, which are usually relying on: 1) growth factors, 2) culture supplements, and 3) some small molecules as neurotrophic factors (Zhang et al., 2017[148]). The higher efficacy of DPSCs in nervous regenerative therapies compared to other MSCs such as BM- or adipose-derived SCs relates to their ability to express higher trophic factors including brain-derived neurotrophic factor (BDNF), glial cell line-derived neurotrophic factor (GDNF), nerve growth factor (NGF), neurotrophin 3 (NT-3), vascular endothelial growth factor (VEGF), and platelet-derived growth factor (PDGF) (Parsa et al., 2010[106]). Also, the higher cytokine expression in DPSCs promotes neuronal differentiation in them (Ullah et al., 2017[131]). The secreted cyto-protective factors help DPSCs to present both direct and indirect neuroprotective properties in nervous diseases and injuries leading to a decreased neurodegeneration in the early stages of pathologies (Parsa et al., 2010[106]; Mead et al., 2013[79]; Ullah et al., 2017[131]). DPSCs have also shown axon regeneration ability even in the presence of axon growth inhibitors in a spinal cord injury model (Parsa et al., 2010[106]) and protection against cell death in an ischemic astrocyte injury model (Ullah et al., 2017[131]).

## Pluripotent Stem Cells from Human Exfoliated Deciduous Teeth

Stem cells derived from human exfoliated deciduous teeth (SHED) or the primary teeth are among the most studied SC types and the most valuable source of stem cells in tissue engineering studies and cell-based regenerative medicine therapies (Martinez Saez et al., 2016[77]). The reason is that these immature SC population advantage from 1) non-invasive isolation procedures, 2) low immune reactions or rejection after transplantation, 3) ability to remain undifferentiated and stable after long-term cryopreservation, 4) being highly proliferative, 5) being easily accessible, 6) potential of multi-lineage differentiation, 7) non-invasiveness, and finally, 8) few ethical concerns (Martinez Saez et al., 2016[77]). SHED can be detected by STRO-1, CD146/MUC18, CD90, CD29, CD44, CD166, CD105, and CD13 (Zhai et al., 2018[146]). Either cultured *in-vitro* or *in-vivo*, SHED populations can successfully differentiate into various specialized cell populations such as odontoblasts, osteoblasts, chondrocytes, adipocytes, and neural cells (Martinez Saez et al., 2016[77]). In 2003, a mixture of SHED and hydroxyapatite/tricalcium phosphate (HA/TCP) was suggested to be used for dental pulp tissue regeneration for the first time (Casagrande et al., 2011[10]). This research was a landmark since dental pulp regeneration is a required step for pulp tissue engineering practices in clinics (Martinez Saez et al., 2016[77]). The SHED mixture has been first implanted in animal models and then was used to proliferate within the scaffold and form dentin-like tissue (Casagrande et al., 2011[10]). Fortunately, SHED has shown potency in adhering to the dentin walls and proliferating within the full-length root canals *in-vitro* (Martinez Saez et al., 2016[77]). In 2008, further advances were achieved regarding the *in-vivo* development of dental pulp from SHED on biodegradable poly-L-lactic acid-based scaffolds, and a pulp-like tissue with a functional vascular network plus odontoblast-like cells substituted the scaffold (Casagrande et al., 2011[10]). The odontoblast-like cells lie on the dentin surface and have an eccentric nucleus and express dentin sialoprotein (DSP) (Casagrande et al., 2011[10]). More recently, SHED was used to proliferate odontoblasts with the expected markers (DSPP, DMP-1, and MEPE) within the full-length root canals on the injected scaffolds *in-vitro* and generate functional dental pulp in the subcutaneous space of mice (*in-vivo*) (Martinez Saez et al., 2016[77]). In this trial, cells could well occupy the root canal space and the tissue regeneration occurred with a promising growth rate (10 μm/day) providing adequate timing for the clinical uses (Martinez Saez et al., 2016[77]; Rosa et al., 2016[116]). The scaffold composition seems to be ineffective on SHED proliferation (Martinez Saez et al., 2016[77]). Tetracycline labeling is a common method for revealing the newly formed dentin. No in-natura study has been conducted to evaluate the SHED potential to proliferate and differentiate in the oral environment (Rosa et al., 2016[116]). 

## Adult Stem Cells

### Induced pluripotent stem cells

Induced pluripotent stem cells (iPSCs) have already shown great promises in animal model trials for regenerative treatment of Parkinson's disease and sickle cell anemia (Jung et al., 2012[53]). Human iPSC-derived MSCs can produce osteoblasts, adipocytes, and chondrocytes *in-vitro* (Wu et al., 2010[137]), and some disease-specific iPSC lines are used for different purposes such as “diseases in a dish” studies (modeling genetic disorders using *in-vitro* induced pluripotent cells), drug developments, and inventing novel therapies (Jung et al., 2012[53]). The iPSCs can also be induced toward vascular and muscle regeneration (Wu et al., 2010[137]). The higher telomerase activity and less senescence of iPSCs-derived MSCs compared to BM-MSCs have introduced them as up-and-coming regenerative alternatives that provide higher survival and engraftment after transplantation (Wu et al., 2010[137]). Despite the extensive suggested applications for iPSCs, their clinical application is not recommended due to the tumorigenesis possibility, which is attributed to the mutagens (e.g., c-Myc) that cause cancers via integration or disrupting tumor suppressor genes (Jung et al., 2012[53]). Another reason for critics of iPSCs applications is the perturbations in epigenetic memories and aberrations in the genomic properties of reprogrammed cells (Jung et al., 2012[53]). Therefore, many precautions are generally recommended while using iPSCs for clinical applications.

## Originated Stem Cells in Dental Regenerative Treatments

### Stem cells from apical papilla

The stem cells from the apical papilla (SCAPs) belong to a unique SC line locating at the apical tissues of the growing tooth roots when at least two-thirds of the root have formed (Lin et al., 2018[66]; Nada and El Backly, 2018[89]). While SCAP derives from the dental papilla, they express a mesenchymal surface marker (STRO-1) and contribute to the epithelial-mesenchymal interactive process of tooth development (Nada and El Backly, 2018[89]). SCAPs can be detected by STRO-1, CD146, and CD24 (Zhai et al., 2018[146]). SCAP has an infection-resistant nature that is explained by its histo-morphologic position concerning the dental pulp (Lin et al., 2018[66]). The apical papilla is separated from the epithelial diaphragm with a cell-rich zone and has access to a collateral circulation that enables the apical papilla to survive a necrotic pulp in just adjacent tissues (Lin et al., 2018[66]). Today, SCAP is readily isolated from the tips of the developing roots of an extracted tooth and treated with a well-established protocol (dissection, digestion using collagenase and protease, and culturing the obtained cell suspensions) to be used for the consequent research or clinical processes (Nada and El Backly, 2018[89]). Compared to DPSCs and PDLSCs, SCAP seems to be a better source to be used in cell-based tooth regeneration because of its higher proliferative and mineralization properties. Primary odontoblasts are mainly differentiated from SCAP during root dentin formation, while replacement odontoblasts are likely derived from DPSCs leading to reparative dentin formation (Zhai et al., 2018[146]).

## Mesenchymal Stem Cells

Mesenchymal stem cells (MSCs) were first obtained from the bone marrow of the iliac crest (Pittenger et al., 1999[110]). However, the term “mesenchymal stromal cells” was attributed to this derivative of adherent cells by the International Society for Cellular Therapy (ISCT) (Horwitz et al., 2005[43]). After their first introduction, their other subsets have also been found in several differentiated tissues such as skin, adipose tissue, and various dental tissues (Egusa et al., 2012[23]). MSCs have a specifically better coating on surface-treated plastic with plasma gas (so-called tissue-culture-treated plates), which makes them distinguishable among all SC types (Horwitz et al., 2005[43]). This type of adult stem cell has shown great promise for clinical applications. Their multipotency of differentiating into osteogenic, adipogenic, and chondrogenic lineages was distinguished long time ago (Pittenger et al., 1999[110]). Furthermore, they are praised for their “stem cell plasticity” feature which enables them to induce similar developing cell lines from typically different origins (Paz et al., 2018[107]). A challenge for the recognition of MSCs has been defining their distinguishing markers among the non-homogeneous populations of adherent cells obtained from the bone marrow (Paz et al., 2018[107]). MSCs are defined by ISCT as tissue-culture-treated plastic adherent stem cells regardless of the tissue from which they are isolated; however, Horwitz et al. have suggested them to be termed as fibroblast-like plastic-adherent cells from any source (Horwitz et al., 2005[43]). 

Emersion and recovery of dental diseases such as deterioration, is substantially under the effect of the teeth microenvironment, so that any pathologic alteration that can affect the endogenous MSCs' functions and their regeneration capacity may lead to substantial bone loss. Similarly, transplanted exogenous MSCs are highly influenced by the microenvironment of both donor and recipient niches that creates a major challenge to using MSCs for therapeutic regeneration purposes in diseased microenvironments (Zheng et al., 2019[153]).

## Extra-Oral Derived MSCs

### Adipose tissue-derived stem cells

Adipose tissue-derived stem cells (ASCs) are considered an abundant MSC source that can be obtained through lipectomy or lipoaspiration from different adipose tissues such as the chin, hips, upper arms, and abdomen (Mizuno et al., 2012[84]). The ASCs eliciting procedure is considered a low invasive process, and they show a robust osteogenesis potency; hence, they can be considered as a promising alternative source of MSCs for periodontal regeneration therapies while their efficiency in guided bone regeneration (GBR) and implant surgery has been demonstrated (Mizuno et al., 2012[84]). 

## Intra-Oral Derived MSCs

### Dental tissue-derived MSCs

Epithelial stem cells and MSC-like cells are reported to locate within particular niches in dental pulp, dental follicle, and periodontal tissues (ligament stem cells) (Egusa et al., 2012[24]; Paz et al., 2018[107]). Up to now, several oral tissues have been introduced as sources for stem cells, including exfoliated deciduous teeth, orofacial bone marrow, apical papilla, dental follicle, dental pulp tissue, periodontal ligaments, oral epithelium, periosteum, salivary glands, and gingival lamina propria tissues (Ercal et al., 2018[26]). These are multipotential cells producing different dental organs such as reparative dentin (Paz et al., 2018[107]). Other tissues differentiated from dental pulp-derived SCs include osteogenic, dentinogenic, adipogenic, chondrogenic, myogenic, and neurogenic cells (Egusa et al., 2012[24]). Periosteum-derived stem/progenitor cells with osteogenesis potential differentiate into osteoblasts and chondrocytes. Their MSC-markers make them suitable candidates for tissue engineering and bone regeneration (Paz et al., 2018[107]).

## Postnatal Human Dental Pulp Stem Cells

Similar to muscles' and nerves' tissues, the dental pulp is a specialized tissue during postnatal life that also contains SCs conferring it the tissue regeneration ability in response to injury (Apatzidou et al., 2018[3]). Dental pulp stem cells (DPSCs) are a subpopulation of mesenchymal cells residing within the pulp tissue that differentiate into odontoblasts during tooth formation under the influence of epithelial and dental papilla cells' interactions (Honda et al., 2010[42]). Generally, the origin and nature of postnatal cells as the precursor of various specialized tooth-associated cell types are little known (d'Aquino et al., 2007[21]). Further characterization of DPSCs has been facilitated by transplanting human DPSCs into immunocompromised mice (Apatzidou et al., 2018[3]). These progenitors are hypothesized to be originated from dental pulp and produce supportive connective tissue and odontoblasts, yet the origin of these cells has not been characterized, and the possible existence of a postnatal DPSC was never heretofore proposed (Apatzidou et al., 2018[3]). Odontoblasts, as one of these precursor cell populations, have a polarized columnar morphology, with eccentric nuclei located at the outer edges of dentin and form reparative dentin in response to general mechanical erosion or disruption (Honda et al., 2010[42]). In 2007, d'Aquino et al. isolated DPSCs acting as odontogenic progenitors from adult dental pulp tissue and early developing dental root tissue, and showed that similar to bone marrow cells, DPSCs could differentiate into odontoblast-like cells and develop mineralized nodules *in-vitro* (d'Aquino et al., 2007[21]). Further studies have identified DPSCs as clonogenic and highly proliferative stem cells especially in animal testing (Apatzidou et al., 2018[3]). The “transplanted” DPSCs produce dentin and pulp-like tissue in much more volume than it would be formed *in situ* during the lifetime of an organism (Apatzidou et al., 2018[3]). 

## Dental Follicle Stem Cells

The dental follicle stem cells (DFSCs) are located within the dental follicle or bilayered Hertwig's epithelial root sheath (HERS); they originate from the ectomesenchymal progenitor cell population and differentiate into cementoblasts or osteoblasts (cementogenesis) during tooth root formation (Honda et al., 2010[42]). DFSCs can be detected by STRO-1, CD90, CD105, nestin, notch-1 (Zhai et al., 2018[146]). DFSCs are discussed in predominant studies to differentiate consecutively into cementum, PDL, and alveolar bone (collectively termed as periodontium), which confirms DFSCs as the origin of cementoblasts (Fawzy El-Sayed and Dörfer, 2016[28]). Therefore, the dental follicle and its containing stem cells exert several critical functions during tooth development, including periodontium development, bone resorption, tooth eruption, osteoclastogenesis and osteogenesis regulation, and proliferation into stratified squamous epithelium under pathological conditions to generate dental cysts (Honda et al., 2010[42]).

## Periodontal Ligament Stem Cells

Human periodontal ligament stem cells (PDLSCs) are a few mesenchymal progenitor cells within the PDL that remain proliferative, and their differentiation potential provides great promises for SC-based regenerative therapies in dentistry (Wang et al., 2010[135]; Bright et al., 2015[7]). PDLSCs can be detected by STRO-1, CD146, CD73, CD90, and CD105 (Zhai et al., 2018[146]). The envisages for clinical use of PDLSCs come from many positive preclinical results in a wide range of *in-vitro* and *in-vivo* studies (Bright et al., 2015[7]). Yet, they are not economically competitive enough to be moved through the commercial usage phase compared to the current root canal therapies and dental implants (Wang et al., 2010[135]). PDLSCs are multipotent cells that can produce structures similar to cementum and periodontal ligament *in-vivo*, which can be used for regeneration of the periodontium in periodontal diseases (Chen et al., 2016[15]). But, the non-lethal nature of periodontitis has caused the periodontal tissues not to be a resolute target for SC-therapy researches (Wang et al., 2010[135]). PDLSCs are reported to be capable of forming a complete periodontal attachment apparatus by generating several periodontal tissues containing multiple developmental lineages such as osteoblasts, fibroblasts, and cementoblasts (Wang et al., 2010[135]; Bright et al., 2015[7]). In periodontal regeneration trials, the affected teeth have been used as ideal models for evaluating new treatments and facilitating surgical-free therapies (Chen et al., 2016[15]).

## Non-Dental Tissue-Derived MSCs

### Gingiva-derived mesenchymal stem cells

Gingival mesenchymal stem cells (GMSCs) originate from gingival connective tissue, mostly referred to as lamina propria (Ercal et al., 2018[26]). The gingiva (both attached and free parts) shows similar immunophenotypic characteristics to MSC-like cells from healthy periodontal tissues and is an accessible source for collecting SCs via a minimally invasive route even in cases of inflamed gingiva, gingival hyperplasia, or periodontal lesions (Apatzidou et al., 2018[3]). The immunomodulatory properties and multipotency for differentiation into various mesenchymal lineages of GMSCs have made them a useful candidate for application in antitumor therapies, skin wound repair, periodontal tendon regeneration, peri-implantitis treatment, bone defect regeneration, as well as treating oral mucositis, experimental colitis, collagen-induced arthritis, and contact hypersensitivity (Fawzy El-Sayed and Dörfer, 2016[28]).

## Salivary Gland-Derived Stem Cells

Salivary gland-derived stem cells (SGSCs) were firstly isolated from a rat submandibular gland (Emmerson and Knox, 2018[25]). Similar to DPSCs, SGSCs can be used as an autologous graft in different procedures, including SC-based dental regeneration therapies as well as head and neck cancer (HNC) (Emmerson and Knox, 2018[25]). SGSCs are multipotent and highly proliferative progenitor cells that can produce acinar, myo-epithelial, and ductal cell lineage markers and can be induced to differentiate into chondrogenic, osteogenic, and adipogenic cells (Emmerson and Knox, 2018[25]). The SGSCs isolated from the human salivary gland can express both embryonic and adult stem cell markers (Emmerson and Knox, 2018[25]).

## Growth Factors

Growth factors (GFs) are natural biological molecules with growth-promoting activities that usually have been initially identified for their functions as mediators and regulators in cellular events (Mercola and Stiles, 1988[81]; Mani et al., 2014[76]). GFs are generally clustered into the superfamilies of epidermal growth factors (EGF), insulin-like growth factors (IGF), transforming growth factors (TGF), heparin-binding growth factors (HBGF), and platelet-derived growth factors (PDGF) (Mercola and Stiles, 1988[81]). Different subclasses in each superfamily have similar structures but multiple functions (Zhu et al., 2017[155]). These scaffolds (such as methylcellulose and β-TCP) play a role as GF delivery vehicle and mechanical support for cell migration and also contribute to the formation of new bone, cementum, and/or periodontal ligament (Kaigler et al., 2011[54]). Although, the direct application of GFs has also been shown that significantly helped to improve retention, bone height increase, and alveolar bone formation to fill osseous defects (Howell et al., 1997[44]). In this regard, several *in-vitro* and *in-vivo* researches imply the mediation of cell chemo-attraction, differentiation, and proliferation in the GFs capability of increasing tissue regeneration capacity (Kaigler et al., 2011[54]). Based on their promising records on the GFs' positive effect on tissue regeneration, GF delivery has been introduced as an assistive strategy in scaffold-based bone tissue regeneration procedures (Özdemir and Ökte, 2012[104]). Despite some promising outcomes of PRP clinical trial treatments, its application is controversial and has not yet gained the acceptable results of β-tricalcium phosphate (β-TCP) in tissue replacement for repairing intrabony defects (Sculean et al., 2007[120]; Özdemir and Ökte, 2012[104]). Next to PRP, GFs are obtained from platelet-rich fibrin (PRF), which is prepared by centrifugation of the natural blood with no additives and contains a fibrin matrix embedding higher content of platelet cytokines, growth factors, and leucocytes (Naik et al., 2013[90]). Another frequent GF-based treatment for bone regeneration following tissue defects is applying enamel matrix derivative (EMD) for stimulating regeneration of the soft and hard tissues surrounding the teeth (Sculean et al., 2007[120]). EMD is an extract of porcine fetal tooth material involved in cementogenesis via stimulating proliferation and growth of fibroblasts while inhibits that of epithelial cells (Ribatti et al., 2011[115]). Considering the extensive current applying of EMD and PRP in periodontal tissue regeneration, identifying their active ingredients and defining the role of their numerous proteins in osteogenesis and cementogenesis can improve the future clinical outcomes (Ribatti et al., 2011[115]). Several studies have targeted identifying the mechanism of these ingredients, including recombinant growth factors (rGFs). The most extensively used rGFs in bone regenerative therapies contain bone morphogenetic protein (BMP)-2, platelet-derived growth factor (PDGF)-BB, and fibroblast growth factor (FGF)-2 (Ribatti et al., 2011[115]). BMP-2, PDGF-BB, and FGF-2 have shown commendable results in bone and periodontal regeneration (Sculean et al., 2007[120]). Therefore, several studies are assessing the capability of different GF combinations for future applications in tissue regeneration therapies. 

## BMPs

Bone morphogenetic proteins (BMPs) are multi-functional growth factors from the transforming growth factor-beta (TGFβ) superfamily (Chen et al., 2004[15]). The growth-promoting function of BMPs includes regulating chondrogenesis and osteogenesis during embryo development (Zhu et al., 2017[155]). Recently, the role of BMPs in the formation of many organs and tissues, especially in teeth and dentin regeneration, is increasingly identified (Zhu et al., 2017[155]). BMPs have proved beneficial for use in tissue engineering, craniofacial complex regenerative therapy, and even regenerating a complete tooth to be used in endodontics and periodontal surgeries for tooth replacement (Kaigler et al., 2011[54]). The applicability of BMPs is for their potential to induce cell proliferation, collagen synthesis, ALP activity, and osteocalcin synthesis in osteoblast cells. Hence, they involve in regulating the expression of the molecular markers (e.g., ALP, DSPP, dentin matrix protein 1, and Nestin) in odontoblasts in human dental pulp, stimulating the odontoblast differentiation, and inducing the formation of dentin-pulp complex (Zhu et al., 2017[155]). BMPs induce morphogenesis through complex signaling networks, which are regulated in three levels of intracellular domains, membrane sites, and extracellular sites (Nakashima, 2005[91]). BMPs' role in inducing DPSCs differentiation into odontoblast-like cells can be conceived from the significant acceleration of the odontoblast differentiation markers' activity (Zhu et al., 2017[155]). Different types of BMPs are extensively used in induction procedures on the embryonic teeth to stimulate their initiation, development, morphogenesis, cell differentiation, and matrix secretion (Nakashima, 2005[91]). Studying the varied expression pattern of the BMP family members among different organisms have revealed their roles in morphogenesis and cell differentiation (Nakashima, 2005[91]). For example, some BMPs are only expressed in a spatiotemporal-specific manner (Bmp2-Bmp7). Three BMP family members are identified in the primary culture of hDPC (Bmp2, Bmp4, and Bmp6), and ten were cloned from the rat incisor pulp tissue (Bmp2, Bmp4, Bmp6, Bmp7, Bmp8, Gdf1, Gdf5, Gdf6, Gdf7, Gdf11, and GDNF), five are expressed during odontoblast differentiation (Bmp2, Bmp4, Bmp6, Bmp7, and Gdf11), and two are expressed during ameloblast differentiation (Bmp4 and Bmp5) (Nakashima, 2005[91]). Such results from related studies have significantly helped the researchers to disclose the contribution and mechanism of various BMP family members in tooth development. These findings have demonstrated that the epithelial-mesenchymal interaction is substantial in the tooth morphogenesis procedure, as epithelial BMP4 is necessary for odontogenesis induction in the mesenchyme (Ou et al., 2015[103]). A combination of Bmp2, Bmp4, and Bmp7 signals contributes to the enamel knot maintenance, epithelium morphogenesis, and patterning of the tooth crown by affecting both epithelial and mesenchymal cells and influencing the initiation of the secondary knots (Ou et al., 2015[103]). These findings in addition to identifying DPSCs, as well as recently available knowledge about the biomaterial scaffolds, have paved the way for using this protein family in designing dental regenerative treatments (Kaigler et al., 2011[54]). Recently, FDA has approved using the recombinant human BMPs in slow-healing fractures to accelerate bone fusion (Kaigler et al., 2011[54]). For regeneration of the pulp in dental therapies, two BMP-based approaches are mainly taken: the *in-vivo* and the *ex-vivo* methods. During the *in-vivo* strategy, natural healing potential of pulp tissue is elevated by injecting BMP proteins or BMP genes, while in the *ex-vivo* strategy, DPSCs are isolated and differentiated in the laboratory using BMP proteins or BMP genes. The induced SCs are then transplanted into the tooth (Kaigler et al., 2011[54]).

## VEGF

The process of new blood vessel formation or angiogenesis in the context of dental pulp treatments is an essential stage for triggering wound healing, especially in direct pulp capping. Accordingly, it is exquisitely regulated by cells releasing the chemotactic factors that organize the transient inflammatory events (Casagrande et al., 2011[10]). The most important regulator of embryonic angiogenesis (vasculogenesis) is the vascular endothelial growth factor (VEGF), which promotes vessel formation from endothelial cells and regulates vascular permeability and pro-angiogenic responses (Mullane et al., 2008[88]). *In-vivo* study of VEGF in immuno-deficient mice has shown its potential for angiogenesis induction and enhances the survival of subcutaneously transplanted dental pulp cells (Mullane et al., 2008[88]).

## FGF

The fibroblast growth factor (FGF) family consists of 18 receptor-binding members that regulate cellular activities (Chang et al., 2013[12]) such as cell proliferation, morphogenesis, and survival in many tissues (Chang et al., 2017[13]). The controlling functions of the FGF family depend on their affinity to FGF-specific receptors (FGFR) as well as their expression level in different tissues (Chang et al., 2017[13]). In tooth formation and regeneration, the FGF and its cognate receptors (FGFR isoforms) organize a reciprocal communication and constitute an oriented regulatory network between the epithelial and mesenchymal compartments (Parsa et al., 2010[106]). The activation of FGFR tyrosine kinases is an example of the FGF/FGFR regulatory axis in tooth morphogenesis (Chang et al., 2013[12]). *In-situ* hybridization technique has shown that four highly homologous genes encode FGFR family isoforms, which differentially express in dental epithelium and mesenchyme (Chang et al., 2013[12], 2017[13]). An example of the spatiotemporal-specific manner of FGFRs is that while FGFR1, FGFR2, and FGFR3 are expressed in different levels in the developing teeth, FGFR4 is not expressed at detectable levels (Chang et al., 2017[13]). Another instance is expressing FGF4, FGF8, and FGF9 in the epithelium acting as proliferation regulator and apoptosis inhibitor in adjacent mesenchymal cells, in contrast to expressing FGF3 and FGF10 in the dental mesenchyme acting as the proliferative stimulator in epithelial cells (Chang et al., 2013[12]). Therefore, the various patterns of expressing FGF isoforms and FGFRs in hDPCs have been shown to act as the regulator of developing adult dental pulp tissue (Chang et al., 2017[13]).

One of the most important members of the heparin-binding protein family is called basic FGF (bFGF or FGF2 or FGF-β) which plays a critical role in the proliferation of neuronal stem cells and hematopoietic and endothelial cells' progenitor (hemangioblasts) in hDPSC, and also increases the ratio of pulp cells in the S-phase (Um, 2018[132]). In contrast, an *in-vitro* study has shown that TGFβ1 induces differentiating the odontoblast-like cells, which implies the difference among functions of various growth factors (Um, 2018[132]). However, for a more detailed view of the exact role of FGFs and FGFRs in dentin regeneration, more studies on the relationship of different genes' expression and cell proliferation and viability are required.

## PDGF

The regenerative potentials of platelet-derived growth factor (PDGF) were discovered in an animal study almost four decades ago, and the most detailed understanding of its therapeutic mechanism in forming bone, cementum, and periodontal ligament has been further studied since then (Lynch et al., 1989[71]). Determining the potencies of PDGF in promoting angiogenesis, inducing cell migration from the surrounding tissue towards the bone deficiencies, and stimulating cell proliferation extended applying PDGFs in osteogenic approaches (Kaigler et al., 2011[54]). PDGF is naturally produced by platelets during clotting and is found in the bone matrix generally in the three forms of PDGF-AA, PDGF-AB, and PDGF-BB (Lynch et al., 2006[72]). Dentin matrix acts as the main reservoir of PDGF-BB, TGF-β1, and other GFs where they are sequestered and stored during *in-vivo* dentinogenesis (Tabatabaei and Torshabi, 2016[123]). PDGF-BB has been most studied for its proliferative effects on different cell types, including BMMSCs (Tabatabaei and Torshabi, 2016[123]). PDGF-BB has also been applied in a β-TCP scaffold, and its regenerative effect on periodontal defects has been proven (Ribatti et al., 2011[115]). Furthermore, its mitogenic effect on cultured human dental pulp cells (hDPC) is augmented in combination with other modulators such as IGF-1 (Tabatabaei and Torshabi, 2016[123]). Concluded from numerous *in-vivo* and *in-vitro* studies on the PDGF mechanism of action, after any tissue injury, the locally released PDGF binds to surface receptors on specific cells and induces their chemotaxis and mitogenesis in the area of injury (Lynch et al., 2006[72]). These studies mainly imply the potent chemotactic and mitogenic effects of PDGF on various cell types including gingival and periodontal ligament fibroblasts, cementoblasts, and osteoblasts (Lynch et al., 2006[72]). There are other bioactive molecules, including noncollagenous proteins (NCPs), which may derive from bacterial acids or decayed dental materials (Tabatabaei and Torshabi, 2016[123]). Their role in dentin repair or regeneration has brought them up as potential candidates for dentin-pulp repair or tissue engineering purposes (Tabatabaei and Torshabi, 2016[123]).

An alternative for soft tissue regeneration induction is the recombinant human platelet-derived growth factor (rh-PDGF), which is FDA-approved for safety and effectiveness for treating chronic foot ulcers in diabetic patients (Wieman et al., 1998[136]). Regarding the considerable potential of rhPDGF for bone regeneration, some preclinical studies have targeted evaluating its potential for human bone regeneration (Nikolidakis and Jansen, 2008[93]). To name one, rhPDGF has been examined for fracture repair in tibial osteotomy in rabbits where it could considerably increase the bone regeneration with adequate biomechanical strength in the repaired tissue (Tabatabaei and Torshabi, 2016[123]). The subperiosteally injection of PDGF resulted in intramembranous bone formation in long bones (Tabatabaei and Torshabi, 2016[123]). Also, the effect of periodic systemic injection of rhPDGF on increasing the bone density in long bones and spine has been displayed by techniques such as DEXA bone density and QCT scans, biomechanical testing, and histological analyses in various studies (Tabatabaei and Torshabi, 2016[123]). The rhPDGF application has been mainly focused on periodontal and peri-implant regeneration (Howell et al., 1997[44]). However, it has also been used for the augmentation of dental implant site (sinus) and horizontal bone, preservation of ridges, and treating the osseous periodontal defects (in combination with IGF) (Howell et al., 1997[44]).

## Markers and Signaling Pathway of Dental Stem Cells

Currently, tooth damage or loss can be treated with filling, implants, or dentures. Both fillers and implants have significantly improved the customers' satisfaction in the past decade. However, these trends are being replaced with natural teeth development and tissue engineering strategies for tooth regeneration at the forefront of researches in the field of dentistry. Development or repairing the tooth using this technique requires scaffold, stem cells, and growth factors. Stem cells differentiated into mature cells type make the tooth (Feng et al., 2016[30]). In the following, more details about the underlying mechanisms and regulation system remodeling are provided. 

## EphrinB2

EphrinB2 and its associated receptors (EphB2 and EphB4) are other members of the RTK family with a proven function in determining the cell fate and directional (migration) or non-directional (motility) cell mobilization during development. Zhu et al. demonstrated an essential role for the EphrinB2-mediated signaling pathways with EphB2 and EphB4 receptors in the osteoblastic differentiation of osteoblasts, MSCs, and PDLSCs. They emphasized the potential of the EphrinB2 ligand/EphB2 and EphB4 receptors of generating a bidirectional (forward and reverse) signaling as a promotor for osteogenic differentiation (Zhu et al., 2017[156]). EphrinB2 (the ligand) and its receptors (EphB2 and EphB4) are all surface proteins; then, for both forward and reverse signaling pathways initiation, the protein-bounded cells should be in direct physical contact (Heng et al., 2018[41]). Association of other molecules such as EphrinB1, EphrinB3, and EphB4 in the DPSCs movements for the differentiation, odontoblasts generation, and tooth repair, and their contribution to the osteogenic/odontogenic differentiation of DPSCs are poorly investigated (Heng et al., 2018[41]).

## Rho/Rho-Associated Protein Kinase and RhoA/Rho-Associated Protein Kinase

The Rho/Rho-associated protein kinase signaling pathway is one of the suggested mechanisms that facilitates repairing the defected teeth by directing DPSCs differentiation into odontoblasts (Yan et al., 2010[141]; Feng et al., 2016[30]). Rho is the upstream protein of the Rho/ROCK signaling pathway and a GTPase from the Ras superfamily which is divided into three subfamilies of RhoA, RhoB, and RhoC. Fatty kinase and serine/threonine kinases are among the downstream effector proteins in this pathway. Rho GTPase promotes cell proliferation, differentiation, movement, and migration in all eukaryotic organisms (Huang et al., 2018[47]). In general, the Rho/ROCK signaling pathway contributes to the cytoskeleton dynamics, vasoconstriction, as well as cell proliferation, differentiation, polarity, cycling, and apoptosis (Feng et al., 2016[30]). MSCs differentiation into chondrocytes, adipocytes, and osteoblasts is also regulated by Rho/ROCK signaling pathway (Huang et al., 2018[47]). The Rho/ROCK signaling pathway has been named after the most studied downstream effector proteins- ROCK. The Rho/ROCK signaling cascade is triggered by directly binding the activated RhoA to the C-terminus of ROCK that causes its activation. RhoA/ROCK signaling mainly involves cytoskeleton reorganization, since the activated ROCK cause cytoskeleton contraction by phosphorylating the myosin and its modulatory proteins (Nour-Eldine et al., 2016[95]). RhoA/ROCK signaling pathway also regulates the cytoskeletal polymerization by which affects the cell polarity and morphology, and consequently the final destination of cell differentiation (e.g. toward osteoblast generation) (Nour-Eldine et al., 2016[95]; Huang et al., 2018[47]). This effect of the triggered ROCK signaling pathway has been shown to increase the Runx2 and osteocalcin expression and prompt BMSCs differentiation into osteoblasts (Huang et al., 2018[47]). On the other hand, inhibition of the ROCK signaling pathway decreases the Runx2 expression level and LIPUS-induced differentiation of MSCs to osteoblasts (Huang et al., 2018[47]).

## Wnt

The Wnt signaling pathway includes a family of 19 secreted markers (in mammals) that contribute to triggering various cellular activities. The Wnt/β-catenin signaling network is among the sequential and reciprocal signaling interactions in the epithelial/mesenchymal axis that involve the development, morphogenesis, and cyto-differentiation of a tooth (Chen et al., 2009[16]). This complex Wnt-based signaling pathway is activated by Wnt protein/Frizzled receptor binding in the canonical pathway which causes β-catenin accumulation in the cytoplasm. After accumulation, the β-catenin translocates to the nucleus and conjoin to some members of the T cell factor/Lymphoid enhancer-binding factor (TCF/LEF) family leading to transcription activation and finally organ development regulation (Miyazaki et al., 2016[83]). β-catenin is a member of the armadillo protein family which plays different roles in the cytoskeleton-cell membrane interactions by acting as linker at the cell-cell junctions in epithelial and endothelial tissues (adherens junctions, desmosomes, and hemidesmosomes). Therefore, they are substantially involved in signal transduction and regulating cell behavior during tissue development. Accordingly, mutational studies in mice models have shown that constitutively expression of an active form of β-catenin in the epithelium prevents complete tooth formation and can create ectopic teeth. Whereas, the inactive β-catenin expression can arrest the tooth formation at the bud stage (Miyazaki et al., 2016[83]).

## Ras

Ras is a small GTPase protein that mediates the Ras-activated signaling pathway, which involves tooth development through not well-recognized mechanisms (Goodwin, 2013[35]). Ras proteins are activated by some upstream effectors/pathways such as receptor tyrosine kinase cascades (RTKs signal), mitogen-activated protein kinase (MAPK), and PI3K pathways. Zheng et al. showed the role of MAPK and PI3K pathways in DESC using the mouse incisor in the DESC regulation and amelogenesis. The rodent incisor is a proper model selected for ectodermal organ renewal and regeneration studies since it grows continuously life-long because it contains large amounts of ESCs and MSCs. Then, they used Ras signaling dysregulations (RASopathies) to show that Ras, MAPK, and PI3K pathways inhibit hyperproliferation, transit-amplification, and enamel formation in DESCs (Zheng et al., 2017[154]).

## cAMP Response Element-Binding /Bone Morphogenetic Protein

The cAMP response element-binding (CREB) protein is a cellular transcription factor that binds to specific DNA sequences called cAMP response elements (CRE). CREB protein is fundamentally involved in both embryonic and adult osteogenesis. One of the modulatory functions of CREB in bone synthesis and metabolism is regulating the osteogenic marker genes such as BMP2 in osteoblasts. The contribution of CREB metabolism in osteogenic activities has been proven in several studies. These studies evidence that small molecules can induce osteoblast differentiation by activating CREB (Miyazaki et al., 2016[83]). However, the exact regulatory mechanisms of CREB on BMPs are not fully understood. Although, the CREB pathway in coordination with some other signaling pathways is hypothesized to upregulate the BMP2 transcription leading to osteoblast differentiation and bone formation (Zhang et al., 2011[150]). Several molecules/pathways are studied and showed to mediate in CREB/BMP2 function in proliferation and odontogenic differentiation, especially in DPSCs. An *ex-vivo* study by Zhu et al. indicated that adrenomedullin (AM) inhibits the apoptosis in DPSCs and regulates BMP2 expression, which promotes the DPSCs differentiation into odontoblast-like cells, and that CREB signaling pathway is involved in this function (Zhu et al., 2017[155]). Another study by Zhang et al. implied the parathyroid hormone (PTH) intervention in the CREB/BMP2 signaling pathway in osteoblastic differentiation (Zhang et al., 2011[150]).

## NF-κB

NF-κB pathway is involved in numerous regulating processes, including tooth organogenesis and eruption (Cai et al., 2011[9]). NF-κB also modulates the tooth development and inflammation in interaction with other processes such as Notch signaling and PI3 K/Akt pathway (Li et al., 2014[64]). It is well recognized that any obstruction in NF-κB pathway causes a developmental arrest of teeth. Several factors such as trauma, inflammatory factors, MTA, TNF-α, and estrogen can trigger the NF-κB pathway in DPSCs, while other factors such as BMS-345541 (a selective inhibitor of IκB kinase that raises the cellular response to inflammation) can inhibit it.

Exposure to a proper cytoplasmic level of TNF-α can cause phosphorylation, ubiquitination, and proteolytic degradation of IκB resulting in the activation of classical NF-κB pathway and translocation of NF-κB to the nucleus, where it can influence on the genes' expression. IκB complex- or the κB inhibitory proteins- is the major regulator of NF-κB that maintains it in a latent form in the cytoplasm. This complex is composed of two catalytic subunits (IKKα and IKKβ) and a regulatory subunit (IKKγ). BMS-345541 selectively blocks the IKKα and IKKβ subunits and downregulates the activity of NF-κB. Li et al. established a canonical NF-κB pathway regulation method in a cellular model. They showed that treating hSCAPs with TNF-α increases the cytoplasmic level of P-P65/P-IκBα. In contrast, treating with BMS-345541 suppresses the expression of P-P65/P-IκBα (Li et al., 2014[64]).

## Regenerative Treatments of Stem Cells in Dentistry

**Figure 1:**
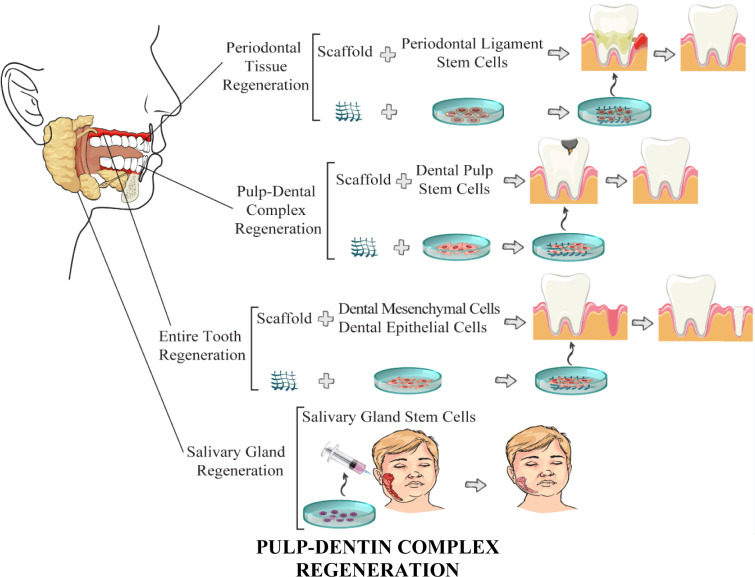
Representation of numerous stem cell-based strategies used in dentistry

Regeneration therapies besides tissue engineering have extensively found their way to the recently trended studies targeted to reproduce and reconstitute a lost or injured organ/tissue to restore its architecture and function to the nearest extent of its original status. Tissue engineering applies a wide range of techniques such as stem cells, biomolecular signaling, and scaffold-based cell cultures to actualize the aim of regeneration therapy in restoring deficient parts (Tatullo et al., 2019[124]). Stem cells have established their situation as a fundamental constituent of this complex strategy for their distinguishing characteristics including; 1) self-reproduction, 2) multipotency, 3) long-term lifetime (Tatullo et al., 2019[124]), and in some cases, their 4) plasticity (Towns and Jones, 2004[128]). The procedures of dental organ regeneration are illustrated in Figure 1(Fig. 1). In the following, every stage is discussed in detail in separate sections. 

The limited efficacy of conventional pulp therapy with durable dental materials has led dentistry to use regenerative alternatives with the ultimate aim of forming mineralized reparative dentin (Tabatabaei and Torshabi, 2016[123]). Today, the restorative healing methods for carious lesions are increasingly becoming attractive for being used instead of surgical removal (ectomy) of diseased tissue in clinical dentistry (Moussa and Aparicio, 2019[87]). Pulpectomy is the most common endodontic treatment, which means total amputation of the dental pulp (pulpotomy), leaving no DPSCs left in dental pulp for later regeneration (Cordeiro et al., 2008[18]). Local regeneration therapy of dentin-pulp complex is a new strategy designed to avoid pulpectomy. This method uses the residual dental pulp followed by pulp amputation to regenerate the dentin-pulp complex (Hashemi-Beni et al., 2017[39]). The pulp amputation is exerted after removal of the decayed coronal pulp tissue and the irrigation of the root canal orifice with chemical reagents (MTA or Ca(OH)_2_-based materials), which promote dentin bridge formation (Morotomi et al., 2015[85]). The inevitable pulp amputation due to appearing layers of necrotic tissue at the residual pulp-dentin bridge interface has led dentistry to develop the local regenerative pulp treatments (Moussa and Aparicio, 2019[87]). For this purpose, researchers found that only vital pulp can stimulate the dentin-pulp complex regeneration; therefore, they used modern tissue engineering to recap the amputated pulp with biomaterial scaffolds, growth factors, and stem cells to promote pulp tissue regeneration including the collateral blood supplement dentin-like hard tissue formation (Hashemi-Beni et al., 2017[39]). This treatment method tries to resemble the cellular procedures involved in soft tissue-wound healing such as chemotaxis, in-site MSC proliferation, and ECM production (Tabatabaei and Torshabi, 2016[123]). Potentially, this method is suggested to be beneficial for various cases including the unexposed cavity preparations for preserving the pulp vitality, exposed pulp cases, and even severely compromised dental pulps (Moussa and Aparicio, 2019[87]). Preserving the pulp vitality is important since the newly constituted dentin bridge is a poorly calcified porous hard tissue that cannot conserve the residual root pulp. On the other hand, the pulp amputation does not promote pulp/dentin regeneration because the blood is only supplied from the root apical meristem which is insufficient (Hashemi-Beni et al., 2017[39]). The very first attempts for SC-based regenerative dentin and pulp tissue therapy failed due to the inability of detecting dental stem cells that could differentiate into odontoblast-like cells (Hashemi-Beni et al., 2017[39]). In 2004, the first MSC populations were identified in the human PDL tissue (Seo et al., 2004[121]). Later, several stem cell lineages from various resources with a suitable performance were discovered which could construct the dentin-like mineral structure and are supposed to be able to restore the lost structure (Hashemi-Beni et al., 2017[39]).

Additionally, dentin-like structures have been experimentally created from human DMSCs (hDMSCs) in immune-compromised mice in several *ex-vivo* studies. According to several *in-vivo* reports, hDMSCs cultured on specific scaffolds can reproduce the vascularized pulp tissue. Also, pilot human trials have proven the potential of DMSCs for safe and efficient regeneration of dental pulp and dentin formation. In this regard, DMSCs are also cultured on particular scaffolds in association with some biomolecules and shown to be effectively able to regenerate the pulp tissue in the pulp chamber following pulp removal (Orsini et al., 2018[99][100]). 

In these SC-based therapeutic strategies that use DPSCs for regenerating dentin-pulp complex, GF concentrations in the dentin matrix should be precisely considered, because they are directly in contact with the pulp and consequently, have a significant effect on DPSCs proliferation as the first stage of pulp repair (Moussa and Aparicio, 2019[87]). The dentin molecules show regulatory influence on the surrounding pulp MSCs through signaling pathways that enhance the healing of injured pulp tissues. Then, understanding their function and interaction with the SCs can greatly help designing more efficient therapeutic strategies (Moussa and Aparicio, 2019[87]). It has been reported that unexposed cavity preparations enhance the residual dentin thickness, which protects the pulp against the detrimental effects of dental materials. Also, in the exposed pulp cases, dentin molecules induce reparative dentinogenesis and help to restore the structural integrity of dentin. Additionally, MSC-based treatments are used to seal the root canal in endodontic therapies for managing severely compromised dental pulps, which is again in direct interaction with dentin materials (Moussa and Aparicio, 2019[87]). The cellular and molecular events in pulp are also affected by caries and trauma causing inflammation and/or regeneration as defense mechanisms for managing the infection (Hashemi-Beni et al., 2017[39]).

## Periodontal Tissues Regeneration

The highly specialized connective tissue surrounding the root of teeth is called periodontal ligament (PDL) that pivotally contribute to embedding the tooth in the jaw, maintaining the tooth homeostasis, repairing, feeding, and also harboring progenitor cell populations called PDLSCs (Seo et al., 2004[121]). PDLSCs derive from mesenchymal stem cells and rarely present in dental follicles mostly in locations near blood vessels and endosteal spaces and have surprisingly shown higher growth potential than BMMSCs and human DPSCs (Seo et al., 2004[121]; Tomokiyo et al., 2019[126]). Although, PDLSCs are considered a highly promising stem cell population; one reason is that their self-renewal capacity has been reported to remain after 100 population doublings, over two times of the human BMMSCs proliferation capacity (Tomokiyo et al., 2019[126]). These cell populations can differentiate into various types of mesenchymal-lineage cells, such as chondrocyte-like cells and adipocyte-like cells containing cytoplasmic lipid droplets with highly expressed adipose-related markers (Xiao and Nasu, 2014[138]). The most useful cell types differentiated from PDLSCs for reparative dentistry are osteoblast-like cells forming Alizarin Red-positive mineralized nodules with high expression of bone-related markers (Seo et al., 2004[121]). The identified markers of human PDLSCs include early MSCs-related cell surface molecules (e.g., STRO-1, CD146/MUC18, CD10, CD26, CD29, CD73, and CD349/FZD9) as well as stromal and endothelial cell-associated surface molecules (CD44, CD90, CD105, CD166, and Stro-3) (Tomokiyo et al., 2018[127]). Some of these markers (e.g., CD44, CD73, and CD90) are similarly expressed in hPDLSCs, hBMMSCs, and human dermal fibroblasts, while some others such as CD146 are detected to be strongly expressed only in PDLSCs, suggesting CD146 as a specific cell surface marker of hPDLSCs (Tsumanuma et al., 2011[130]). The hypoxia condition, Rho-kinase signaling pathway, and mechanical loading are among factors that cause the high self-renewal capacity and proliferation rate of PDLSCs (Tomokiyo et al., 2019[126]). Another factor that fundamentally affects the PDLSCs' regenerative capacity is the scaffolds on which they are transplanted. The human PDLSCs have been firstly transplanted on a scaffold from HA/TCP in animal models (Izumi et al., 2011[50]) and successfully resulted in the reconstitution of cementum- and PDL-like structures (Seo et al., 2004[121]). This positive result helped other researchers to overcome their hesitance for using PDLSCs in other animal models (e.g. dog, rat, mice, etc. of either wild-type, immuno-deficient, or with various artificial defects in periodontium) on various biomaterials as scaffold (e.g. **β**-tricalcium phosphate/type I collagen, gelatin sponge, HA disks, etc.) and successfully achieve regenerated periodontium tissues (i.e. cementum, collagen fibers, and nerve fibers) (Tsumanuma et al., 2011[130]; Han et al., 2014[38]; Ninomiya et al., 2014[94]). These results suggested that either allogeneic or autologous grafts of PDLSCs, dental bud cells (DBCs), molar, etc. can form bone-, cellular cementum-, dentin/pulp-like complex structures (containing odontoblast-like cells and blood vessels in the pulp), and PDL-like tissues with bone-related markers (e.g. ALP, OPN, BSP, and OCN) if transplanted with optimized initial cell concentration and suitable GFs/scaffolds (Kuo et al., 2008[61]; Tsumanuma et al., 2011[130]). The transplants have been usually grafted in artificially periodontium defects (Tsumanuma et al., 2011[130]; Han et al., 2014[38]); however, in some studies, the fascia of the dorsal muscles, adult renal capsules, or jaw bone (both maxilla and mandible) have also been a destination for different types of dental stem cells (Kuo et al., 2008[61]; Ninomiya et al., 2014[94]). As an instance, in Ninomiya et al.'s study, PDLSCs were grafted in the fascia of the dorsal muscles of wild-type Lewis rats, and forming matrices by PDLSCs in the HA disks was proven by immunohistochemical staining (Ninomiya et al., 2014[94]). In another allogeneic transplantation trial, human PDLSCs were cultured on an HA/TCP scaffold and transplanted into the dorsal region of immuno-deficient mice with the most common calcium phosphates. In the end, the PDL-, bone-, cementum-, and surprisingly Sharpey's fiber-like tissues (between PDL and cementum) were successfully replaced and augmented in the generated complex (Park et al., 2011[105]).

## Cementum Regeneration

Hertwig epithelial root sheath (HERS) cells are considered to be cementogenetic and able to produce a thin layer of acellular cementum around the root neck. HERS also can cover the lower part of the root by thicker cellular cementum. A challenge in periodontal regeneration researches using cellular intrinsic fiber cementum (CIFC) is achieving an adequate attachment function. The low-quality attachment of the newly-formed cementum in CIFC is due to lack of acellular extrinsic fiber cementum (AEFC), low density of the inserting fibers, and weak interfacial tissue bonding (Liu et al., 2019[67]). In damaged periodontal tissues, some cementum-specific proteins are recognized to be responsible for promoting the new cementum and bone formation via inducing specific signaling pathways. These pathways promote mitogenesis, raise the cytosolic level of Ca^2+^, and actuate the protein kinase C (PKC) cascade. These mechanisms lead to the migration and preferential adhesion of progenitor cells. The cementum-specific proteins (CEMPs) associated with these activities contain cementum-derived growth factor (CDGF), cementum attachment protein (CAP), and cementum protein-1 (CEMP1). The CEMPs' activity induces the differentiation of periodontal ligament stem cells (PDLSCs), dental follicle stem cells (DFSCs), and, adipose-derived stem cells (ADSCs) into cementoblasts and osteoblasts resulting in periodontium regeneration, PDL fibers formation, periodontal angiogenesis, and creation of a cementum-like mineralized extracellular matrix (ECM) (Liu et al., 2019[67]). 

## Salivary Glands Regeneration

Regeneration of the salivary gland has been a topic of interest, especially in HNC studies principally through two general regenerative approaches. In the related researches, the salivary glands' function has been tried to be re-established either via constructing and grafting an artificial salivary gland by tissue engineering or via transplanting stem cells in the damaged salivary tissue. MSCs and BMSCS are the most stem cells applied for restoring the functionality of the damaged salivary glands (Ogawa and Tsuji, 2017[98]). The presence of multiple stem/progenitor-like cells in the salivary glands has been shown using different techniques such as genetic lineage tracing, DNA labeling, *in-vitro* floating sphere assays, and two-dimensional (2D) or 3D cultures of salivary gland cells (Lombaert et al., 2017[69]). The SGSCs can be identified and isolated using expression detection of some specific markers, including cell surface receptors and cytokeratins at different times during organ development (Paz et al., 2018[107]). The occasionally appearing of stem/progenitor cells in reservoir compartments accounts for maintaining the organ homeostasis- even in the adult salivary glands- and also for organ development via compensating the cell loss by duplication, maintenance, and/or expansion (Lombaert et al., 2017[69]; Paz et al., 2018[107]). 

## Entire Tooth Regeneration

Entire-tooth loss can lead to several physical and mental sufferings which may extensively compromise the life quality and self-esteem of the patient. Even, in some wildlife species, losing the complete dentition can end their life. The regeneration of the entire tooth has come to the focus of many pieces of research after successful results of regenerating tooth elements. As a major organ, a tooth is constructed from multiple hard and soft tissues. Enamel, dentin, and cementum are the hard tissues that surround the dental pulp as the only vascularized tissue in teeth (Balic, 2018[4]; Morsczeck and Reichert, 2018[86]). Generally, new teeth formation is exerted through two main strategies of recombined tooth germs and tooth-shaped scaffolds. The first one is developed using DMSCs and DESCs, and transplanted into the alveolar bone to grow further and erupt from the gingiva. The second one is also filled with DMSCs and DESCs assumed to finally form a functional tooth after being implanted into the alveolar bone (Orsini et al., 2018[99][100]). 

During several trials for regenerating teeth, several disassociated osteoprogenitor cells from porcine or rat were cultured in collagen, PLGA, or silk-protein biomaterial scaffolds and yielded putative dentin and enamel organ. For such complex interdisciplinary regenerative approach cells, biomaterial scaffolds, and signaling factors are the key factors that should be measured elaborately because of their central roles. A significant objective of related trials has been to achieve the stem/progenitor cell regeneration within the body without being necessarily manipulated *ex-vivo* (Kuo et al., 2008[61]). A relevant achievement was attained by Kuo et al. who auto-grafted a mixture of unerupted DBCs with bone marrow fluid on a gelatin/chondroitin-6-sulfate/hyaluronan tri-copolymer (GCHT) scaffold into the original alveolar sockets of the pigs and observed putative tooth-like tissues, alveolar bone, and periodontal ligament, as well as complete tooth at least in one animal (Kuo et al., 2008[61]). Before Kuo's study, it was revealed that the bone marrow fluid (containing growth factors and morphogens secreting BMSCs), embryonic oral epithelium, and adult mesenchyme could promote tooth regeneration and up-regulate odontogenic genes (Kuo et al., 2008[61]). In some cases, the regenerated dental structures were ectopic, in indiscriminate shapes, or much smaller than the normal teeth in the same host (Kuo et al., 2008[61]; Botelho et al., 2017[6]). In a comparative study between deciduous and adult DPSCs, both were cultured in a self-assembling peptide-amphiphile hydrogel. As result, deciduous cells showed a higher proliferative rate, while adult cells showed a greater osteogenic differentiation potential (Botelho et al., 2017[6]). In another trial for regenerating the entire tooth, DPSCs and dentin matrix protein-1 were delivered with collagen scaffolds to dentin slices in mice where they could form an ectopic pulp-like tissue (Kim et al., 2010[59]). Also, in a study, seeding the deciduous dental pulp SCs in matrigel within the cross-sectional tooth slices and ectopic implanting them in SCID mice resulted in forming vascular pulp-like tissue (Cordeiro et al., 2008[18]; Kim et al., 2010[59]). Then, a vascularized pulp-like tissue was regenerated using ectopic implantation of SCAPs and dental pulp in root fragments of SCID mice. However, this finding is scientifically valuable, using DPSCs for dental pulp regeneration is difficult to be commercialized and clinically established (Cordeiro et al., 2008[18]). Considering the extensive diversity of tissues that are being regenerated, various doctrines of using cells, biomaterial scaffolds, and signaling molecules are implemented for tissue engineering, but no individual doctrine can be recommended to govern all types of tissue engineering. In the following, the available principles of using cells, biomaterial scaffolds, and signaling molecules in tooth regeneration are reviewed.

## Cell Homing in Tooth Regeneration

Cell homing is a suggested approach as an alternative to cell transplantation for tooth regeneration due to the indispensable barriers associated with the translation of cell-delivery-based tooth regeneration approaches into therapeutics. In all cell-based therapies, including for dental tissue regeneration purposes, both cell source and cell delivery encompass translational hurdles. The other preclusion for the significant clinical translation of tooth regeneration to date is the excessive cost of commercialization and difficulties in regulatory approval (Kim et al., 2010[60]; Yuan et al., 2011[144]). Therefore, cell homing is getting more attraction among the current recognized approaches in tissue regeneration. Omitting the stages of cell isolation and *ex-vivo* cell manipulation makes cell homing more easily adjustable and more potential to be commercialized for clinical processes. Therefore, this under-recognized strategy can be offered as an alternative to cell-delivery-based tooth regeneration (He et al., 2017[40]; Ruangsawasdi et al., 2017[118]). Cell homing has represented to be a potential approach for tooth regeneration showing efficient recruiting of sufficient cells of multiple lineages into scaffold's micro-channels, regenerating a putative PDL, and forming new alveolar bone. In the first study reporting *de novo* formation of tooth-like tissues by cell homing, Kim et al. (2010[59][60]) used the stromal-derived factor-1 (SDF1) for its potential of chemotaxis induction and binding to CXCR4 receptors of multiple cell lineages. They also used the bone morphogenetic protein-7 (BMP7) for its ability to induce elaborating mineralization in dental pulp cells, fibroblasts, and osteoblasts. The combination of SDF1 and BMP7 could induce angiogenesis and acceptably recruit both bone marrow stem/progenitor cells and endothelial cells, which are critical for tooth regeneration within a reconstructed scaffold (Kim et al., 2010[59][60]). Integration of the newly regenerated PDL and bone into the native alveolar bone confer a promising ground for a novel therapeutic approach, which is translatable to the clinics (Yuan et al., 2011[144]). 

## Therapeutic Challenges of Stem Cells in Dentistry

The most commonly used SC types for orofacial bone regeneration have been BMSCs from the iliac crest, DPSCs, ASCs, periosteum-derived stem cells, and osteoprogenitor cells, respectively, that have been applied besides other MSCs derived from different dental tissues (d'Aquino et al., 2009[20]). Stem cell-based regenerative therapy is an under-developing technique for orofacial bone regeneration; then, the whole procedures and events during and following transplantation are not recognized (Meijer et al., 2007[80]). However, the successes obtained in completely restoring human mandible bone defects using DPSCs and a scaffold from collagen sponge have been much promising (d'Aquino et al., 2009[20]). However, more detailed investigations are required to determine the real mechanism of newly-formed bone in SC-mediated ridge augmentation (osteo-induction by transplanted cells or osteo-conduction by host osteogenic cells), since that seeded BMSCs could also release GFs that induce the host cells and act as an osteogenic cell source for new bone formation (Meijer et al., 2007[80]; Pieri et al., 2010[109]). Such a distinction can be noticed using histomorphometric and micro-CT analyses to prove that the cell-based regenerated bone derives from the transplanted cells (Pieri et al., 2010[109]). The retention of cellular osteogenic capacity and viability are other requisites for producing ECM in bone tissue engineering (Pieri et al., 2010[109]). However, the perspective of the clinical outcome of transplanted cells and their immunological consequences are poorly studied. Although a growing amount of evidence state that dental stem cells can be useful for immune and regenerative therapies (Yamaza et al., 2010[140]), migration and dying of transplanted cells have been reported in some animal model studies (Pieri et al., 2010[109]). Surprisingly, hDPSCs show more acceptable results compared to BMSCs for regeneration. SHED are also potential candidates for dental stem cell-based regenerative therapy (Tirino et al., 2011[125]). These SC populations have made a promising future for stem cell-based tissue engineering as a solution for replacing a missing tooth. New dental SC-based treatments are also suggested to be useful for correcting cleft palate sparing, injured teeth, jawbones, periodontal defects, and also biologically *de novo* regeneration of the whole tooth structures (Prabhu and Issrani, 2014[112]). *De novo* regeneration of the whole tooth is the final goal in dentistry and is expected to be the biggest challenge in the relevant future clinical researches (Jamal et al., 2011[51]). However, no restorative material can imitate all physical and mechanical properties of the dental tissue; its regeneration is considered beneficial for improving physiologic dentin deposition and decreasing interfacial damages and the following problems (Volponi et al., 2010[134]). In craniofacial regenerative biology, periodontium regenerative therapeutic strategies have also been highly preferred; however, there are limitations with using autologous bone grafts, allografts, or alloplastic materials in some clinical applications (Jamal et al., 2011[51]). Regarding the ideal therapeutic approach in dental tissues, being the entire tooth regeneration after tooth loss, DESCs and DMSCs have been used together to produce tooth germs that are transplantable into the recipient alveolar bone, where they can proliferate, bud, and convert into a functional tooth (Ikeda and Tsuji, 2008[48]). In this regard, dental bud cells have been grafted in the PRF scaffolds in the miniature swine model (Wang et al., 2010[135]). Another approach for de novo tooth regeneration has been implanting a complex of tooth-shaped polymeric biodegradable scaffold filled with DESCs and DMSCs; however, up to now, it has been only tried in ectopic sites and the grafting material has not formed neither an acceptable crown morphology nor an accomplished root (Oshima and Tsuji, 2014[101]). Despite, very latter researches are implemented in mice models showing that having a functional teeth with entire roots is possible (Oshima and Tsuji, 2014[101]), and the entire dental tissue can be obtained permitting the de novo regenerated teeth to erupt and completely integrate into the ground alveolar bone (Otsu et al., 2014[102]). 

## Immune Modulation

MSCs have immunosuppressive effects on the innate immune system by inflammatory factors production inhibition or anti‐inflammatory secretion induction through direct cell-cell contact or releasing soluble factors such as PGE2, sHLA-G, TGF‐β, IDO, and HGF (Brown et al., 2011[8]). MSCs can also inhibit macrophage transformation into the M2 type, which produces anti‐inflammatory factors such as IL‐10 and TGF‐β, instead of M1 type that produces inflammatory factors such as IL‐1β, TNF, IL‐6, IL‐12, IL‐18, and reactive oxygen species (ROS) such as NO and superoxide (Cui et al., 2016[19]). On the other hand, MSCs suppress NK cells, inhibit the expression of activating NK-cell receptors, decrease IFN-**γ** secretion, and promote cytotoxic effects on the virus-infected cells (Cui et al., 2016[19]). Their inhibitive function is mediated by secreting factors (such as TGF-β, sHLA-G, IDO, and PGE-2), direct cell-cell contact via MSCs' surface TLR-4, and suppressing the secretion of NKp30 and NKG2D (NK-cell activation-related surface receptors). MSCs decrease the IL‐12 secretion and expression of dendritic cell (DC) maturation surface markers (CD80, CD86, MHC‐II, and CD11c), and produce TSG‐6 which prevent DCs from stimulating T‐cell responses. All of these inhibitory mechanisms prevent DCs from maturation and function. MSCs also inhibit DC maturation by releasing PGE2 that causes DCs to fail in producing IL-12 and prevent inducing T-cell to produce GM-CSF and IL-4. They also stimulate monocyte transformation to immature dendritic cells (IDCs). Additionally, MSCs inhibit the formation of neutrophil extracellular traps (NETs) by preventing neutrophils from activation and oxidative burst and releasing hydrolytic enzymes such as peroxidase and protease. MSCs also inhibit mast cell activation and degranulation and suppress the pro-inflammatory pathways through the COX-2-dependent mechanism (Brown et al., 2011[8]). MSCs carry out their immunosuppressive function on B-cells and T-cells in the adaptive immune system by secreting enormous immunosuppressive factors, chemokines, and adhesion molecules. These factors suppress effective T-cell by intervening in the procedures of T-cell proliferation, differentiation, T-cell mediated cytotoxicity, and programmed cell death stimulated by nonspecific mitogens, co-inhibitory molecules of B7-H4 and HLA-G, Fas/Fas ligand pathway, etc. (Laing et al., 2018[62]). MSCs can also prevent B cells from differentiation into plasma cells and decrease IgM and IgG1 production (Feng et al., 2014[29]). MSCs can also disrupt the IFN secretion by T-cells and BAFF secretion by DCs, resulting in the suppressed B-cell proliferation and function (Zhang et al., 2019[147]). MSCs also induce TH1 transition into TH2 by declining the IFN-γ secretion in TH1 pro-inflammatory cells and raising IL-4 secretion in TH2 anti‐inflammatory cells (Zhang et al., 2019[147]). 

## Conclusion

Regenerative dentistry is increasingly recognized as a state-of-the-art field of medicine among dental clinicians during dental treatments as a procedure of acquiring stem cells (from deciduous teeth, third molars, gingiva, etc.) and storing them for potential future autologous treatments. Adult stem cells have been successfully acquired from sources such as oral and maxillofacial regions. For achieving the ultimate purpose, which is craniofacial regeneration, there has remained a long way to be paved for identifying the effective factors in immunomodulatory functions of adult MSCs, BMSCs, and pluripotent stem cells. Such information is required for a more effective outcome of SC-based bone and periodontal tissue restoration, especially for transplanting at the inflamed sites. Knowing the immunomodulatory properties of adult stem cells used in dental and periodontal regeneration is vital for reaching optimal tissue regeneration and controlling the local immune responses during transplantation. Among different strategies in the regenerative dentistry field, tissue engineering and chair-side cellular grafting approaches are more promising because of their more predictable regenerative results. The randomized controlled type of clinical trials with long follow-ups is the most required type of scientific evidence for a comprehensive establishing of reconstructive dental therapeutics. Another necessary field to be elaborated is understanding the biological processes underlying both graft donor and recipient during bone regeneration. Despite all unrecognized aspects of stem cell-based bone and periodontal tissue reconstruction, prosthodontists are increasingly being attracted to stem cell biology because of its successful results as well as the unresolved inefficiencies of implant dentistry. 

## Future Direction

The mechanisms that control the fates and functions of the transplanted stem cells need to be studied. Despite multiple SC-based studies on the dental pulp and periodontal regeneration in animal models, serious considerations, and clinical trials with long-term follow-up are necessary to speed up the translation of basic and preclinical SC-based studies to the dental clinics in terms of regulation, immunity, technology, ethics, and any other possible clinical restriction. These fundamental concerns need to be sorted out to make regenerative treatments practically applicable and beneficial for patients with pathological or injured dental pulp and/or periodontal tissues. Autologous stem cells are already started to be used in some clinical trials for regenerating pulp and periodontal tissues; however, their approval as well as outcomes have not yet been broadcasted and transmitted to the guidelines or indications. Stem cell-based regenerative approaches can help many people around the world who suffer from dentistry complications that strongly warrant further studies. Fortunately, the technologies of modern imaging systems, nanotechnology, and mathematical modeling are increasingly developing and coming to help stem cell-based regenerative studies achieve more reliable and qualitative outcomes in a much shorter time. 

## Notes

Mohsen Yazdanian and Hamid Tebyanian (Research Center for Prevention of Oral and Dental Diseases, Baqiyatallah University of Medical Sciences, Tehran, Iran; Phone: +989198045743, Fax: +982182482549, E-mail: tebyan.hamid@yahoo.com) equally contributed as corresponding authors.

## Authors’ contribution

The authors declare that this work was done by the persons named in this article. Armin Soudi, Mohsen Yazdanian, Reza Ranjbar, Hamid Tebyanian, and Alireza Yazdanian were involved in study design and data collections. Armin Soudi, Mohsen Yazdanian, Reza Ranjbar, Hamid Tebyanian, Elahe Tahmasebi, Ali Keshvad, and Alexander Seifalian were involved in critically reviewing the data and writing the review article.

## Acknowledgement

The authors would like to acknowledge the useful comments were given by colleagues at the Research Center for Prevention of Oral and Dental Diseases, Baqiyatallah University of Medical Sciences, Tehran, Iran.

## Funding

There was no financial support.

## Ethical approval

This article is a review and does not contain any experiment on humans or animals performed by any of the authors.

## Conflict of interest

The authors declare that they have no competing interests. 

## Figures and Tables

**Table 1:**
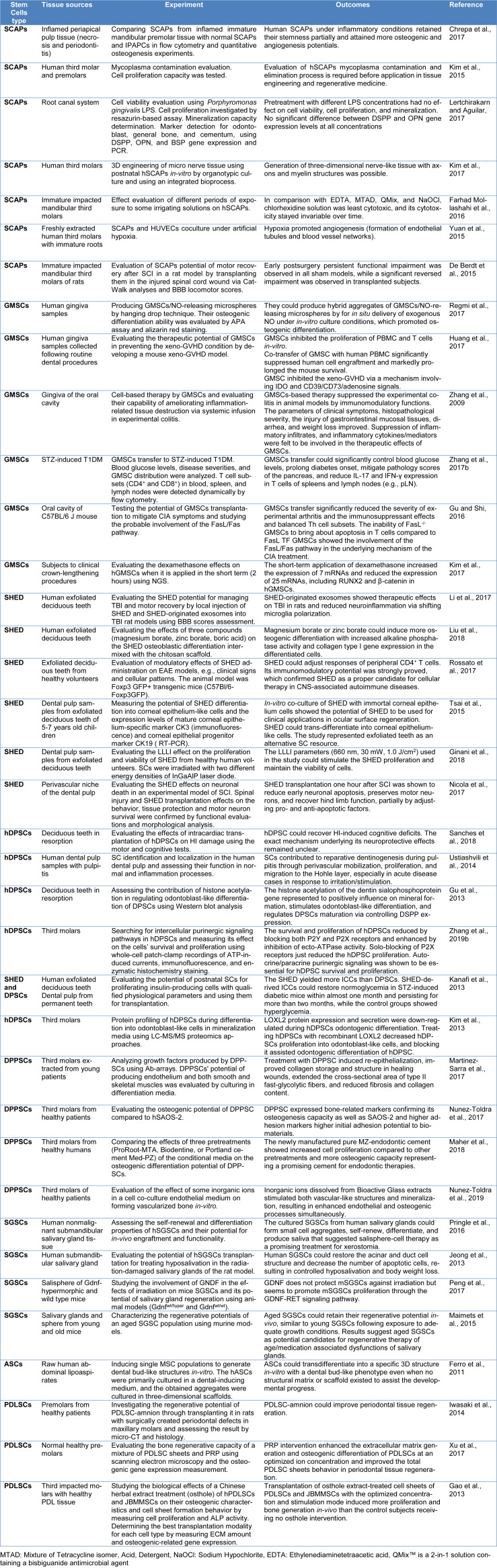
Applications of oral stem cells in regeneration
